# Optimization of Microfluidic Preparation for Calcium Alginate Microspheres Based on Response Surface Methodology

**DOI:** 10.3390/bioengineering13101144

**Published:** 2026-09-30

**Authors:** Na Li, Yuanbo Gao, Liang Lu, Yu Liu, Zhenbin Zhang, Xing Zhang

**Affiliations:** 1 College of Life Science and Engineering, Shenyang University, Shenyang 110044, China; 2 Institute of Metal Research, Chinese Academy of Sciences, Shenyang 110016, China; 3 School of Materials Science and Engineering, University of Science and Technology of China, Shenyang 110016, China

**Keywords:** calcium alginate microspheres, microfluidics, response surface methodology

## Abstract

Although calcium alginate microspheres prepared by microfluidic methods have been widely investigated, the combined effects of dispersed-phase composition, continuous-phase properties, and surfactant combinations on microsphere formation remain insufficiently understood. In this study, a microfluidic system was established using sodium alginate as the aqueous phase, mineral oil as the continuous phase, and calcium chloride as the receiving phase. Response surface methodology was applied to model and optimize the key parameters involved in microsphere formation. The regression model showed good fit and reliable predictive ability. Under the optimized conditions, validation experiments yielded an average target-size fraction of 81.25%, which agreed well with the predicted value, demonstrating the feasibility and reproducibility of the optimized process. Biological evaluation showed that the optimized alginate microspheres exhibited no significant cytotoxicity toward BxPC-3 cells and maintained high cell viability, as confirmed by Live/Dead staining and CCK-8 assay. Confocal imaging further showed that the encapsulated BxPC-3 cells remained highly viable and formed three-dimensional clusters within the alginate matrix. These findings provide a reproducible microfluidic approach for generating calcium alginate microspheres with potential utility in cell encapsulation, three-dimensional culture, and related biomedical applications.

## 1. Introduction

Calcium alginate microspheres have considerable application potential in cell entrapment, three-dimensional culture and tissue engineering because of their excellent biocompatibility, mild ionic cross-linking behavior, and strong encapsulation and protective capacity [1,2]. Although conventional methods, including spraying, mechanical stirring-based emulsification and electrostatic droplet generation, can be used to prepare alginate particles, they often produce broad size distributions and show limited repeatability and poor process stability. These limitations make it difficult to achieve the level of control required for microencapsulation. Microfluidic technology enables precise control over droplet generation, interfacial stability, and mass transfer processes in microscale environments, allowing continuous production of droplets with controllable particle sizes and excellent dispersibility, thereby providing a novel technical pathway for the controlled preparation of calcium alginate microspheres. Droplet generation in microfluidic chips can be regarded as a microscale emulsification and pinch-off process governed by interfacial tension, viscous shear, and flow-rate matching between the dispersed and continuous phases. Based on the properties of the two phases, it can be divided into types such as water in oil, oil in water, and multiple emulsification [3]. Therefore, according to the above microfluidic droplet formation mechanism, sodium alginate solution is dispersed into monodisperse droplets through microchannels, and then collected in a calcium chloride solution, where rapid ionic crosslinking occurs to form structurally stable calcium alginate microspheres [4].

Although microfluidic technology has been applied in the preparation of calcium alginate microspheres, when applied in the cellular field, it is necessary to coordinately regulate multiple factors, including dispersed-phase composition, continuous-phase properties, and surfactant type and concentration, in order to balance droplet-generation stability, target-size fraction, and cell compatibility [5,6,7]. The choice of continuous phase directly affects the stability of droplet formation and cell activity. Common continuous phases include liquid paraffin, fluorinated oil, mineral oil, etc., which have their own characteristics in viscosity, hydrophobicity, gas permeability and cost [8].

In some cell-encapsulation studies, paraffin oil has been used as the continuous phase because of its convenient handling and acceptable biocompatibility. Rossow et al. [9] successfully prepared monodisperse Saccharomyces cerevisiae cell-loaded microspheres using paraffin oil as the continuous phase in the microfluidic cell embedding experiment. The mild gelation was achieved by thiol-ene click reaction without free radicals. Finally, the survival rate of yeast cells was as high as 99%, indicating the biocompatibility and operational stability of paraffin oil in cell microencapsulation. Lu et al. [10] systematically evaluated the emulsifying behavior of silicone oil as a continuous phase in the simulated intraocular environment, and found that the addition of high-molecular-weight polydimethylsiloxane (HMW PDMS, 423 kDa) can significantly improve the tensile viscosity of silicone oil and inhibit its adhesion and emulsification on the surface of cell coating, and found that the rheological properties of the continuous phase have an important impact on the stability and biocompatibility of the biological interface.

Ahmed et al. [11] used fluorinated oil Novec^TM^ 7500 as continuous phase, adding fluorine-containing surfactant Pico-Surf^TM^. The monodisperse and uniform calcium alginate microspheres were prepared without chelating agent and pH change, which was conducive to maintaining the activity of embedded cells and enzyme function. Although fluorinated oils are widely used in droplet microfluidics, they may not be the most practical choice for the present study, which relies on external Ca^2+^-mediated gelation and cost-efficient process optimization [12]. The formation of calcium alginate microspheres depends on the controllable diffusion of Ca^2+^ to aqueous droplets. Fluorinated oil is almost insoluble in calcium salts, which must rely on aqueous phase premixing or complex double emulsion droplet structure, significantly increasing the complexity and uncontrollability of the experimental system [13,14,15]. The price of fluorinated oil is relatively expensive. For experiments requiring large-scale parameter optimization, fluorinated oil is no longer feasible as a consumable for conventional experiments, whereas mineral oil is substantially less expensive than fluorinated oil and is therefore more suitable for large-scale parameter screening.

Previous studies have shown that non-fluorinated oils, including mineral oil, peanut oil and grape seed oil, were systematically evaluated for microfluidic cell encapsulation. With appropriate surfactants, all tested continuous phases supported high viability (>90%) of human colorectal adenocarcinoma cells. Mineral oil, however, maintained stable droplet formation across a wide flow-rate range because of its higher viscosity, low cost and ready availability [16,17,18]. The effects of mineral oil, olive oil and paraffin oil on droplet formation behavior in a flow-focusing microfluidic device were investigated using combined numerical simulation and experimental analysis [19]. The results show that when the continuous phase flow rate is constant, the droplet size formed by mineral oil (surface tension 0.0520 NGM, dynamic viscosity 0.105 PA·s) is the largest, and the fluctuation of droplet size is the smallest when the flow ratio changes, showing good stability and roundness.

In addition to the continuous phase properties, the two-phase interfacial tension and fluid viscosity also play a key role in the formation and stability of droplets in the microchannel [20,21]. Appropriate surfactant addition can reduce interfacial tension, promote uniform droplet formation, and suppress droplet coalescence through the interfacial self-assembly of amphiphilic molecules [22]. The hydrophilic–lipophilic balance (HLB) value is commonly used to guide surfactant selection; for example, in W/O systems, Span 80 and Tween 80 can be combined at different ratios and concentrations to regulate interfacial properties and droplet formation behavior [23].

However, systematic optimization studies focusing on the coupled effects of dispersed-phase composition, continuous-phase properties, and surfactant parameters in microfluidic preparation of cell-compatible calcium alginate microspheres remain limited [24,25]. The single factor optimization method cannot fully reflect the interaction between the factors, and cannot accurately construct the quantitative relationship between process variables and microspheres formation efficiency, which restricts the optimization and large-scale application of the preparation process of calcium alginate microspheres with cell compatibility. Response surface methodology (RSM) is a statistical method suitable for multi-factor and multi-level process optimization. It can evaluate the main effect and interaction effect of each factor on the response value under less test times, and establish the regression model between factor and response. RSM was applied to optimize the microfluidic controlled preparation process of calcium alginate microspheres, which was helpful for quantitative analysis, so as to improve the systematicness of process optimization and the repeatability of results. Based on this, a microfluidic controlled preparation system of calcium alginate microspheres was constructed with sodium alginate as the aqueous phase, mineral oil as the continuous phase and calcium chloride as the receiving phase. Using microspheres formation efficiency as the primary response, Response Surface Methodology was employed to model and optimize key process parameters, analyze the effects of individual factors and their interactions on microspheres formation, and evaluate in vitro cell viability. This study aims to establish a stable and reproducible microfluidic process for the preparation of calcium alginate microspheres and to provide an experimental basis for their further application in cell encapsulation, three-dimensional culture, and related biomedical fields.

## 2. Materials and Methods

### 2.1. Design of Microfluidic Droplet Generation Device

**Figure 1:**
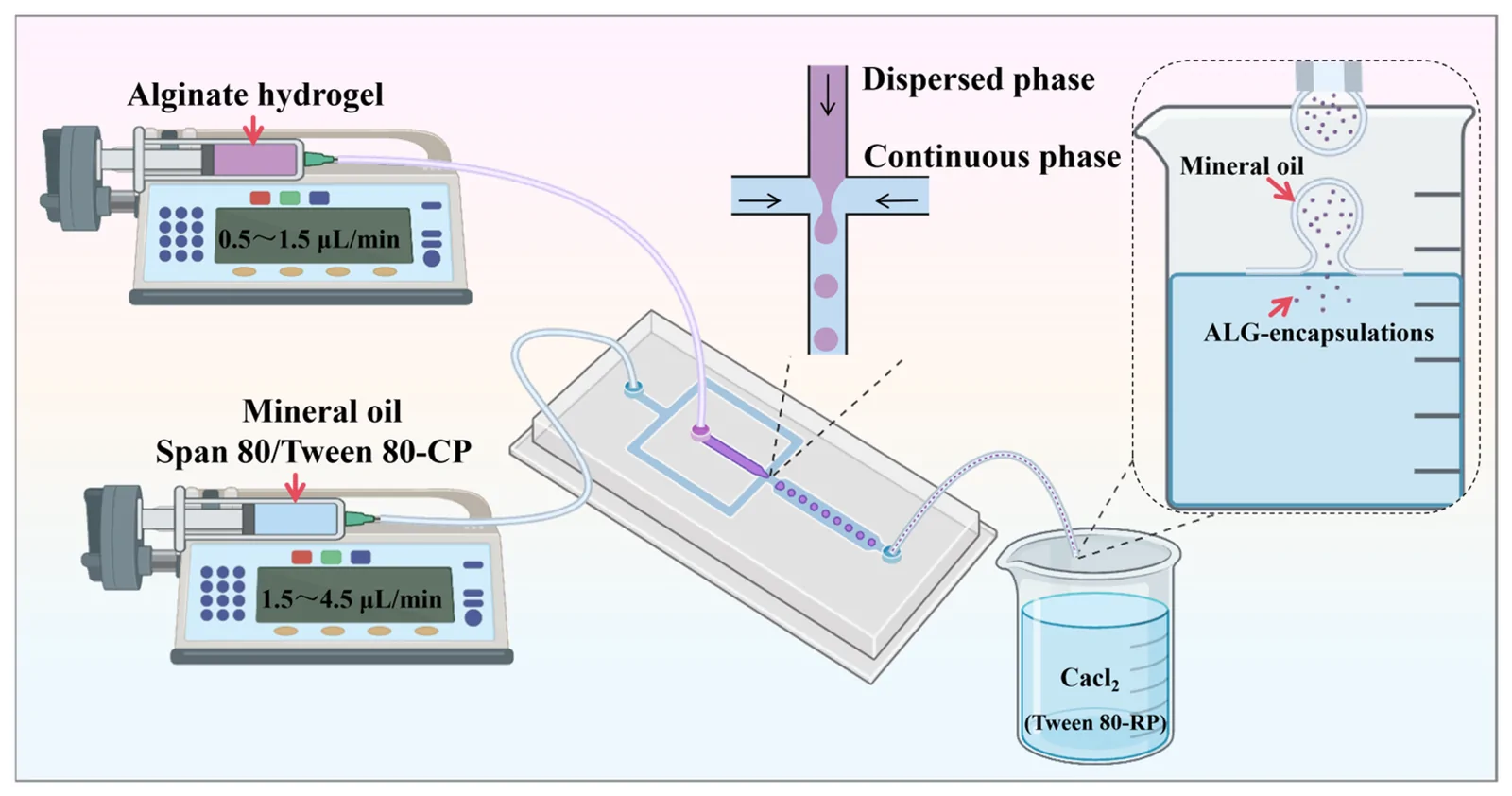
Schematic illustration of the microfluidic droplet generator.

As shown in Figure 1, a flow-focusing microfluidic platform for droplet generation was designed and constructed in this study. The device was equipped with two syringe pumps (WH-SP-02 dual-channel syringe pump; Suzhou Wenhao Microfluidic Technology Co., Ltd., Suzhou, China) for delivery of the continuous and dispersed phases, and Span 80 was added to the continuous phase as a surfactant. A flow-focusing glass-bonded PDMS microfluidic chip (Suzhou Wenhao Microfluidic Technology Co., Ltd.) was utilized for droplet generation in this study. The chip is configured with two inlet channels for the continuous phase and dispersed phase respectively and one collection outlet, and all microchannels have a uniform depth of 50 μm and width of 200 μm. The droplet formation process is independently controlled by two syringe pumps. The oil phase flow rate is 1.5~4.5 μL/min, and the water phase flow rate is 0.5~1.5 μL/min. Notably, the flow rates of the continuous and dispersed phases were maintained as constant process parameters in this study, since our work focused on elucidating the impacts of material formulation on microsphere formation and properties. We placed the chip on the microscope stage, connected the syringes of each phase, and eliminated the bubbles in the pipeline. The droplet formation occurred in a two-phase flow consisting of a continuous phase and dispersed phase. The microchannel was provided with an outlet. After the droplet was formed at the focusing port, it was directly collected into the calcium chloride solution through the outlet channel for solidification, gently stirred, and kept stationary for 15 min to complete the crosslinking. After centrifugation, the collected microspheres were washed three times with PBS to remove the residual continuous phase and surfactant.

### 2.2. Methods for Experimental Optimization

#### 2.2.1. Single-Factor Screening Experiments

The effects of sodium alginate concentration (0.5%, 1%, 1.5%, 1.8% and 2%), mineral oil as the continuous phase, and surfactant concentration on the formation efficiency of calcium alginate microspheres were investigated separately. The surfactant variables included Span 80 (1%, 2%, 3%, 4%, 5% and 6%), Tween 80 in the continuous phase (0.1%, 0.5%, 1%, 1.5% and 2%), and Tween 80 in the receiving phase (0.1%, 0.5%, 1%, 1.5% and 2%).

#### 2.2.2. Response Surface Methodology

The core principle of Response Surface Methodology (RSM) is to construct mathematical equations and expressions with optimal goodness of fit for a given optimization objective, predicated on experimental or simulated datasets. Box–Behnken Design (BBD) is a widely recognized, classic experimental design protocol within the RSM framework. This design completely eliminates experimental runs at extreme factor levels, enabling a safer and more efficient experimental layout within the predefined range of factor levels, and is particularly suitable for the preliminary optimization of process conditions and the systematic investigation of multi-factor interactions [26].

Based on the results of prior single-factor experiments, the suitable working ranges of four key process variables were determined in this study: sodium alginate concentration of 1.0–1.5% (*w*/*v*), Span 80 concentration in the continuous phase of 3.0–4.0% (*v*/*v*), Tween 80 concentration in the continuous phase (Tween 80-CP) of 0.5–1.0% (*v*/*v*), and Tween 80 concentration in the receiving phase (Tween 80-RP) of 1.0–2.0% (*v*/*v*). Statistical analysis of the experimental data obtained from BBD was performed using Design-Expert software (Version 13.0.5, Stat-Ease Inc., Minneapolis, MN, USA). A quadratic polynomial regression model was subsequently established, and the effects of each investigated factor on the response (the target-size fraction) were evaluated. Based on the results, a quadratic regression equation (1) was fitted to characterize the relationship between the response variable (the target-size fraction, Y) and four independent variables: sodium alginate concentration (A), Span 80 concentration in the continuous phase (B), Tween 80 concentration in the continuous phase (C), and Tween 80 concentration in the receiving phase (D). In this equation, *β*_0_ is the intercept term, *β*_i_ the linear coefficients, *β*_ii_ the quadratic coefficients, and *β*_ij_ the interaction coefficients.

(1) $$Y=\beta_{0}+\sum_{i=1}^{4}\beta_{i}X_{i}+\sum_{i=1}^{4}\beta_{ii}X_{i}^{2}+\sum_{i<j}\beta_{ij}X_{i}X_{j}$$

Analysis of variance (ANOVA) was performed to evaluate the overall significance of the model, the lack of fit, and the significance of individual coefficients. The software was used to generate response surface plots and contour plots and to analyze interaction effects to visualize the effects of the investigated factors on the target-size fraction, as well as the interaction effects between factors. Finally, the process parameter combination yielding the highest target-size fraction was identified by model optimization, and validation experiments were performed to verify the predictive accuracy and reliability of the model. The target-size fraction was quantitatively determined using a combination of inverted microscopy imaging and image analysis. The detailed procedure is as follows: an aliquot of the prepared microsphere suspension was collected, mixed gently, pipetted onto a watch glass, and observed and imaged under an inverted microscope (Olympus CX43, Olympus Corporation, Tokyo, Japan) with a 10× objective lens. Subsequently, the acquired images were analyzed using ImageJ 1.8.0 software, and the diameters of microspheres in each field of view were measured. Microspheres with diameters < 100 μm or >300 μm were excluded according to the preset criteria [27]. The percentage of microspheres with diameters in the 100–300 μm range relative to all counted microspheres was calculated. The target-size fraction (%) = (Number of microspheres with diameters within the 100–300 μm range/Total number of microspheres) × 100%. All experiments were performed in triplicate, and the results are presented as mean values.

### 2.3. Cell Culture and Viability Assays

Human pancreatic cancer cells (BxPC-3), obtained from the Cell Bank of the Chinese Academy of Sciences (Shanghai, China), were seeded into 96-well plates at a density of 4000 cells per well and cultured in RPMI 1640 medium supplemented with 10% fetal bovine serum (FBS) and 1% penicillin/streptomycin at 37 °C in a humidified incubator containing 5% CO_2_. After 24 h, the culture medium in the experimental group was replaced with calcium alginate microspheres containing 10% FBS, while the control group was cultured in conventional RPMI 1640 medium supplemented with 10% FBS. BxPC-3 cells were incubated with calcium alginate microspheres, and on 1, 3, and 5 days, 10 μL of CCK-8 solution (Sigma) was added to each well. The plates were then returned to a humidified incubator at 37 °C with 5% CO_2_ for 3 h. Absorbance at 450 nm was measured using a microplate reader (Thermo Fisher Scientific, Waltham, MA, USA) to evaluate cell viability in the 96-well plates.

Cells were seeded into confocal dishes at a density of 1 × 10^4^ cells/mL. After 24 h, the original culture medium was removed and replaced with medium containing calcium alginate microspheres for co-culture. The cells remained attached to the plate bottom throughout the experiment, and the microspheres were dispersed in the culture medium, making contact with the upper surface of the cell layer. No cells were encapsulated within the microspheres in this set of experiments. This was followed by 48 h of co-incubation of BxPC-3 cells with calcium alginate microspheres, with cell-only as the control condition. The medium containing the calcium alginate microspheres was aspirated, and the cells were gently washed three times with PBS. Calcein AM/PI staining working solution was then added to fully cover the cells, followed by incubation for 20 min. After three further gentle washes with PBS to remove the staining solution, the cells were observed and imaged under an inverted fluorescence microscope.

BxPC-3 cells were encapsulated in calcium alginate microspheres and cultured, followed by live/dead staining and z-stack imaging using a laser scanning confocal microscope to assess the status of the encapsulated cells. Briefly, BxPC-3 cells were added to alginate solution at a density of 1 × 10^8^ cells/mL, and BxPC-3-loaded calcium alginate microspheres were generated using the established microfluidic microsphere fabrication system. The prepared microspheres were further co-cultured for 3 days in an incubator containing 5% CO_2_. Calcein-AM and PI staining solutions were mixed and gently added to the culture dish to completely cover the samples, followed by incubation for 20 min at room temperature. The staining solution was then removed, and the samples were washed three times with PBS for 5 min each to reduce background fluorescence. The stained samples were scanned and imaged using a laser scanning confocal microscope. Calcein-AM and PI were excited at 488 and 543 nm, respectively. Z-stack imaging was performed at 1 μm intervals along the z-axis across the microspheres and adjacent regions, with the scan depth covering the diameter of the microspheres. Serial optical sections were acquired for three-dimensional reconstruction.

### 2.4. Statistical Analysis

The experimental data involved in this experiment were all expressed in mean ± SD. The data between groups were analyzed by one-way ANOVA, and the Tukey HSD post test was further used when significant differences were detected (*p* < 0.05). All data processing, statistical analysis and visualization work are completed using R software (version 4.3.3).

## 3. Results and Discussion

### 3.1. Single Factor Experimental Results and Analysis

#### 3.1.1. Effect of Span 80 Concentration on the Target-Size Fraction

In the preparation of alginate microspheres using the O/W single-layer emulsion method, the surfactant concentration in the continuous phase governs the characteristics of the oil–water interface and interfacial tension. As such, it indirectly affects the capillary number (Ca)—the primary governing parameter for microfluidic droplet formation [28]—and serves as an important factor of both the stability of the primary emulsion and target-size fraction [29,30]. However, at low surfactant concentrations, the interfacial film lacks sufficient strength, making the droplets more prone to coalescence or deformation and thereby reducing target-size fraction. By contrast, excessively high surfactant concentrations may increase system viscosity, render the interfacial film overly rigid, and even give rise to cytotoxicity [31].

**Figure 2:**
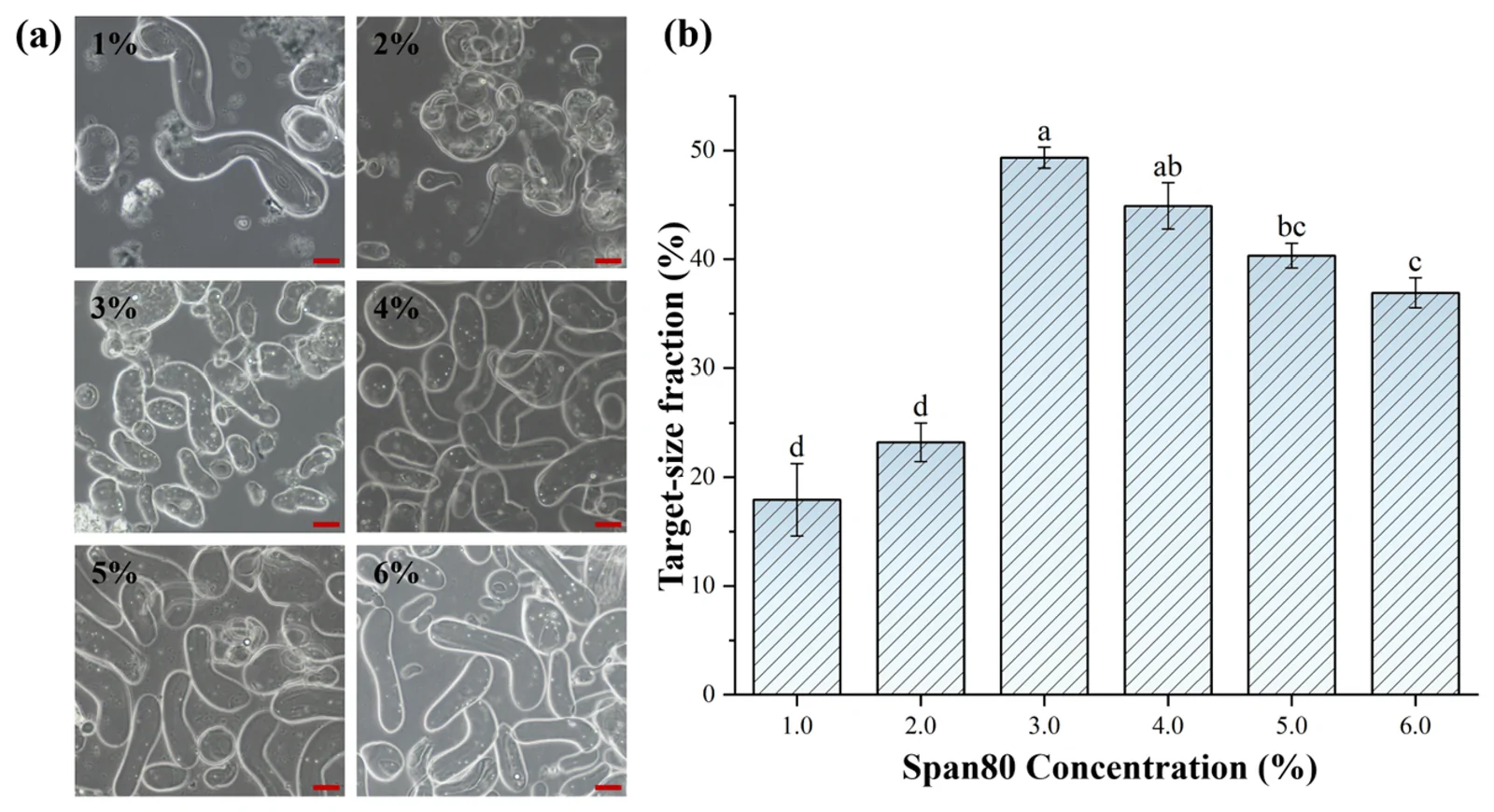
Effect of Span 80 concentration on target-size fraction. (**a**) Optical micrographs of microfluidic-fabricated microspheres, Scale bar = 100 μm; (**b**) corresponding quantitative statistics of target-size fraction (columns with the same letter are not significantly different, *p <* 0.05).

As a lipophilic surfactant, Span 80 adsorbs at the oil–water interface, thereby reducing interfacial tension, facilitating the dispersion and homogenization of dispersed-phase droplets within the continuous phase, and inhibiting droplet polymerization before they enter the curing bath [32]. Based on these results, mineral oil was selected as the continuous phase, and the effect of Span 80 concentration (1%, 2%, 3%, 4%, 5%, 6% (*v*/*v*)) on microsphere formation was investigated. Figure 2a shows that at 1% and 2% Span 80, microsphere formation was poor and no discernible microspheres were observed. When the Span 80 concentration reached 3% or higher, discrete particles became visible, although many exhibited rod-like or oval morphologies. These findings suggest that increasing the Span 80 concentration improved particle formation, but further optimization was required to obtain uniformly shaped microspheres. Previous studies indicate that increasing shape diversity can better recapitulate the complex. Rod-like or fibrous microspheres can be fabricated with high precision using droplet microfluidic technology. Following implantation or injection, non-spherical microspheres can induce cells to align, migrate, and differentiate along a preferred direction, which is critical for constructing regenerative scaffolds for oriented tissues such as muscle, nerve, and tendon [33,34,35]. ImageJ was used to quantify the acquired images for statistical analysis (Figure 2b). As the Span 80 concentration increased, the microsphere formation rate initially rose and then declined, reaching its maximum at 3% (49.35 ± 0.96%). This value was significantly higher than that at 2% (23.21 ± 1.78%; *p* < 0.05). Considering the formation of droplets in the microfluidic chip and the fact that higher Span 80 concentrations can mitigate droplet coalescence during calcification, a Span 80 concentration of 3% was selected for subsequent experiments.

#### 3.1.2. Effects of Tween 80 Concentration in Continuous and Receiving Phases on Target-Size Fraction

**Figure 3:**
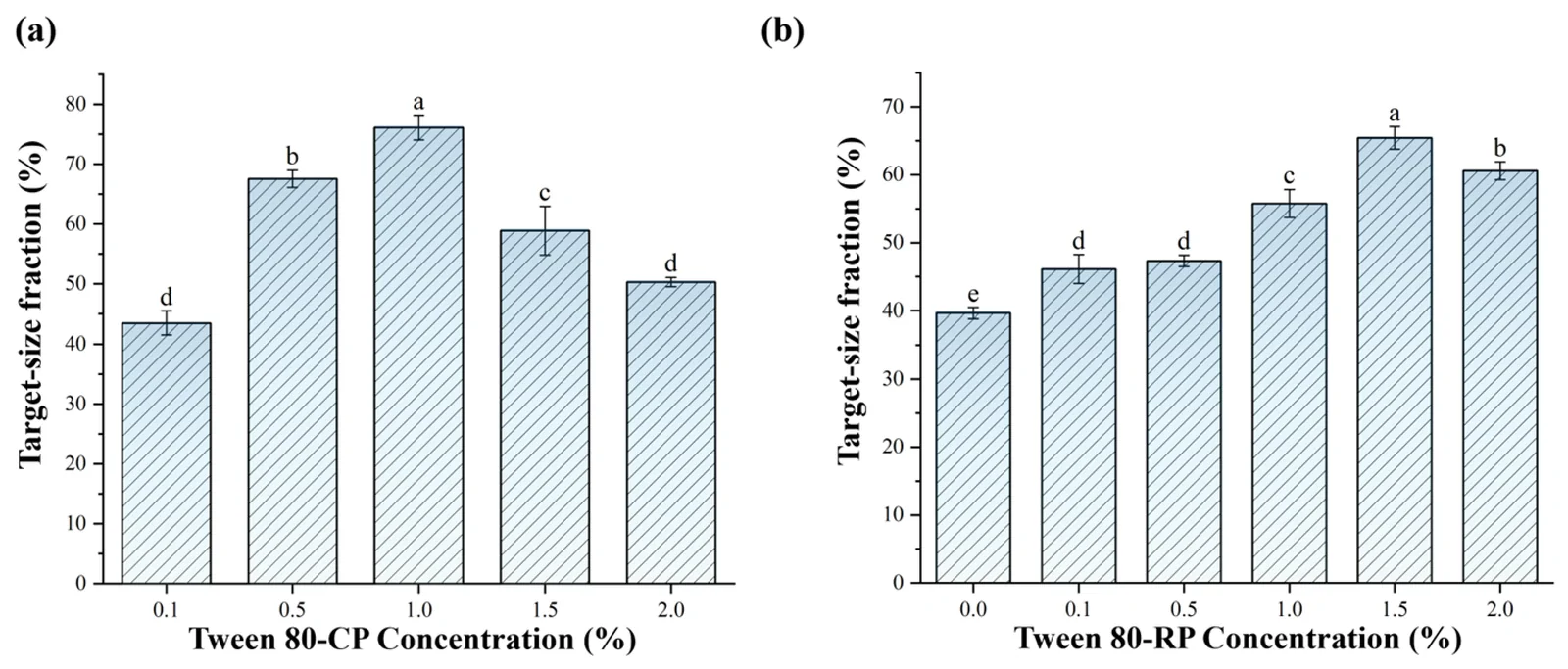
Effects of Tween 80-CP and Tween 80-RP concentrations on the target-size fraction. (**a**) Quantitative statistics of target-size fraction for Tween 80-CP; (**b**) quantitative statistics of target-size fraction for Tween 80-RP (columns with the same letter are not significantly different, *p <* 0.05).

The hydrophilic–lipophilic balance of surfactants in the continuous phase is a key parameter governing microsphere morphology and yield. Tween 80, a hydrophilic non-ionic surfactant, is often used in combination with the lipophilic surfactant Span 80 [36]. Together, they enable cooperative tuning of the HLB at the oil–water interface, thereby significantly influencing emulsion stability and the droplet monodispersity achieved in the microfluidic chip [37]. The inclusion of Tween 80 enhances the fluidity of the interfacial film and inhibits droplet polymerization [38]; however, its concentration must be precisely controlled. At insufficient levels, it cannot effectively counteract interfacial tension, whereas excessive levels may induce phase inversion or render the interfacial film overly loose, ultimately compromising the formation efficiency of microspheres [39]. Therefore, with mineral oil as the continuous phase and the Span 80 concentration held constant, the effects of varying Tween 80-CP concentrations in the continuous phase (0.1%, 0.5%, 1.0%, 1.5%, and 2% (*v*/*v*)) on microsphere molding quality were investigated (Figure 3a). At a Tween 80-CP concentration of 1.0%, the rate of microsphere formation was 76.12 ± 2.08%, significantly higher than those observed at other concentrations (*p* < 0.05). Therefore, 1.0% Tween 80-CP was selected as the optimal concentration for subsequent experiments. In addition, the interfacial properties of the receiving phase (such as surface tension, viscoelasticity, and ionic strength) directly influence how droplets spread, penetrate, and deform at the contact interface. These effects ultimately determine the microspheres’ final target-size fraction, sphericity, and structural integrity [40]. To regulate the wettability and interfacial tension at the oil–water interface, an appropriate amount of the hydrophilic surfactant Tween 80 is added to the receiving phase. This addition promotes the smooth and uniform entry of droplets into the curing bath and also influences the diffusion of calcium ions into the microspheres [41,42]. The effect of Tween 80 RP concentration in different receiving phases (0%, 0.1%, 0.5%, 1%, 1.5%, 2% (*v*/*v*)) on the microsphere formation rate was investigated. The aim was to determine the optimal Tween 80 RP concentration for assisting curing and improving the integrity of the microspheres. As shown in Figure 3b, the rate of microsphere formation increased with rising Tween 80-RP concentrations. At 1.5% Tween 80-RP, the rate reached 65.43 ± 1.65%, which was significantly higher than the rates at adjacent concentrations. Therefore, 1.5% Tween 80-RP was chosen for subsequent experiments.

#### 3.1.3. Effect of Sodium Alginate Concentrations on the Target-Size Fraction

**Figure 4:**
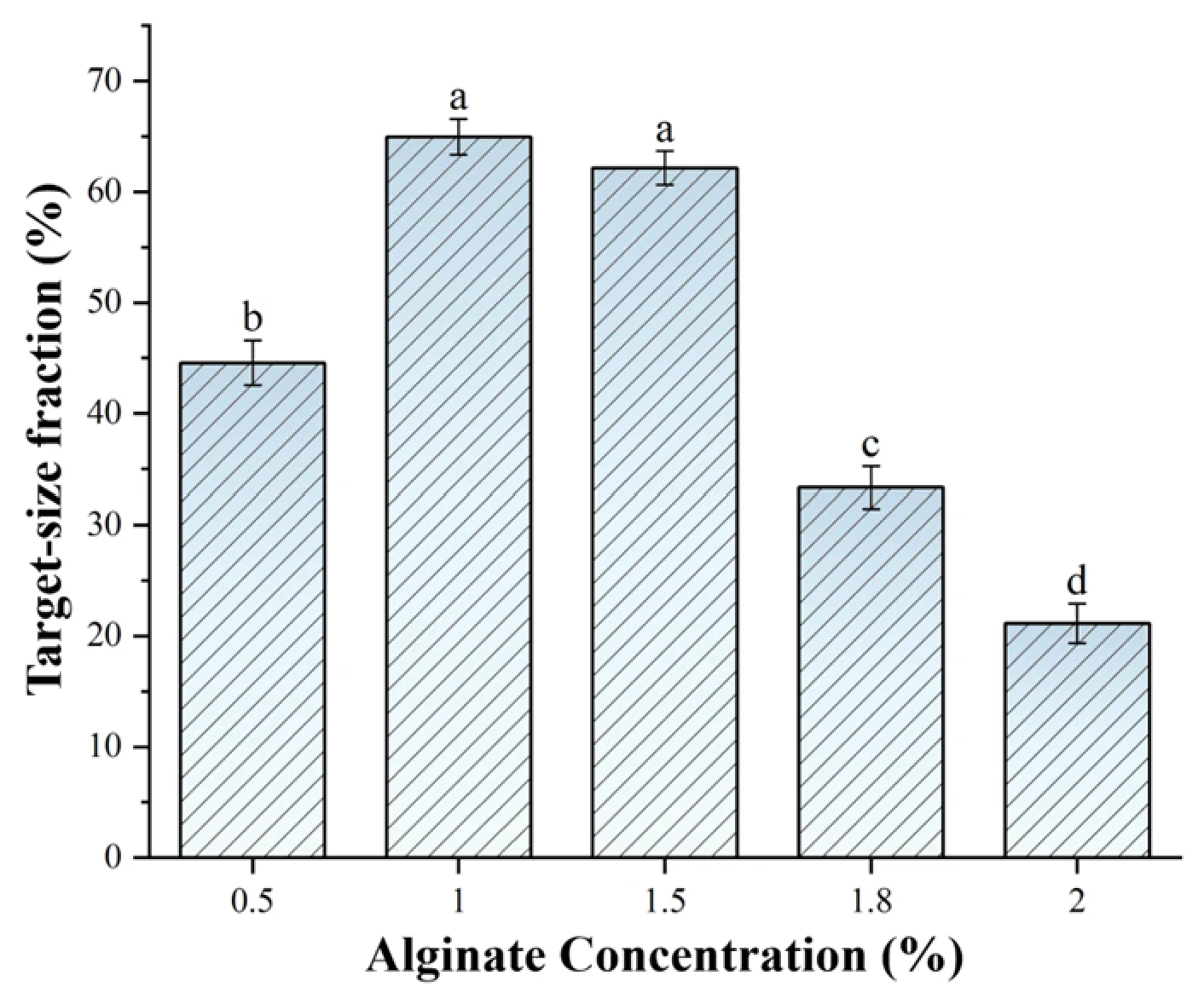
Effect of sodium alginate concentration on the target-size fraction (columns with the same letter are not significantly different, *p <* 0.05).

In a sodium alginate (ALG)-based microencapsulation system, ALG serves as the primary structural polymer, and its solution concentration is the key parameter determining the microspheres’structural integrity and function [43]. This concentration directly influences solution viscosity, gelation kinetics, and the density of the final crosslinked network, thereby playing a decisive role in droplet stability in the continuous phase, the mechanical strength of cured microspheres, and their permeability [44]. A complex trade-off exists between concentration and target-size fraction: at low alginate concentration, the gel network is fragile and cannot maintain structure, whereas high concentration makes the solution overly viscous, resulting in uneven or trailing droplet formation [45]. Therefore, studying the effect of sodium alginate concentration on target-size fraction is essential for optimizing the microsphere preparation process and ensuring batch consistency. Using the defined continuous phase, we designed a single-factor experiment to test sodium alginate concentrations of 0.5%, 1.0%, 1.5%, 1.8%, and 2.0% (*w*/*v*). The effect of each concentration on the target-size fraction distribution was evaluated to determine the optimal concentration. The results (Figure 4) showed that the target-size fraction was significantly higher at 1% and 1.5% sodium alginate than in the other groups. The target-size fraction was highest at 1%, likely because the lower concentration makes the dispersed phase easier to be sheared into droplets by the continuous phase. Higher sodium alginate concentrations yielded microspheres with increased rigidity. Given that sufficient mechanical strength is needed to preserve structural integrity, a 1.5% sodium alginate concentration was selected for further optimization, since it achieved a target-size fraction not significantly different from the maximum observed.

### 3.2. Box–Behnken Experimental Design: Results and Analysis

Based on the factors and levels from single-factor screening (Table 1), a Box–Behnken response surface design with four factors at three levels was generated using Design-Expert software. In this design, the target-size fraction was the response variable, and the sodium alginate microsphere production process was modeled and systematically optimized, resulting in 29 experimental runs. The experimental design and results are summarized in Table 2, and the regression model’s ANOVA is presented in Table 3.

Experimental data were fitted to a quadratic (second-order) polynomial response surface model; the resulting model Equation (2) for the response variable is as follows:

R = +81.80 + 3.79 × A − 1.51 × B + 0.8317 × C + 2.47 × D − 0.2275 × A × B − 2.80 × A × C + 2.96 × A × D + 9.57 × B × C + 1.43 × B × D + 3.35 × C × D − 18.29 × A^2^ − 22.04 × B^2^ − 17.42 × C^2^ − 14.76 × D^2^ (2)

**Figure 5:**
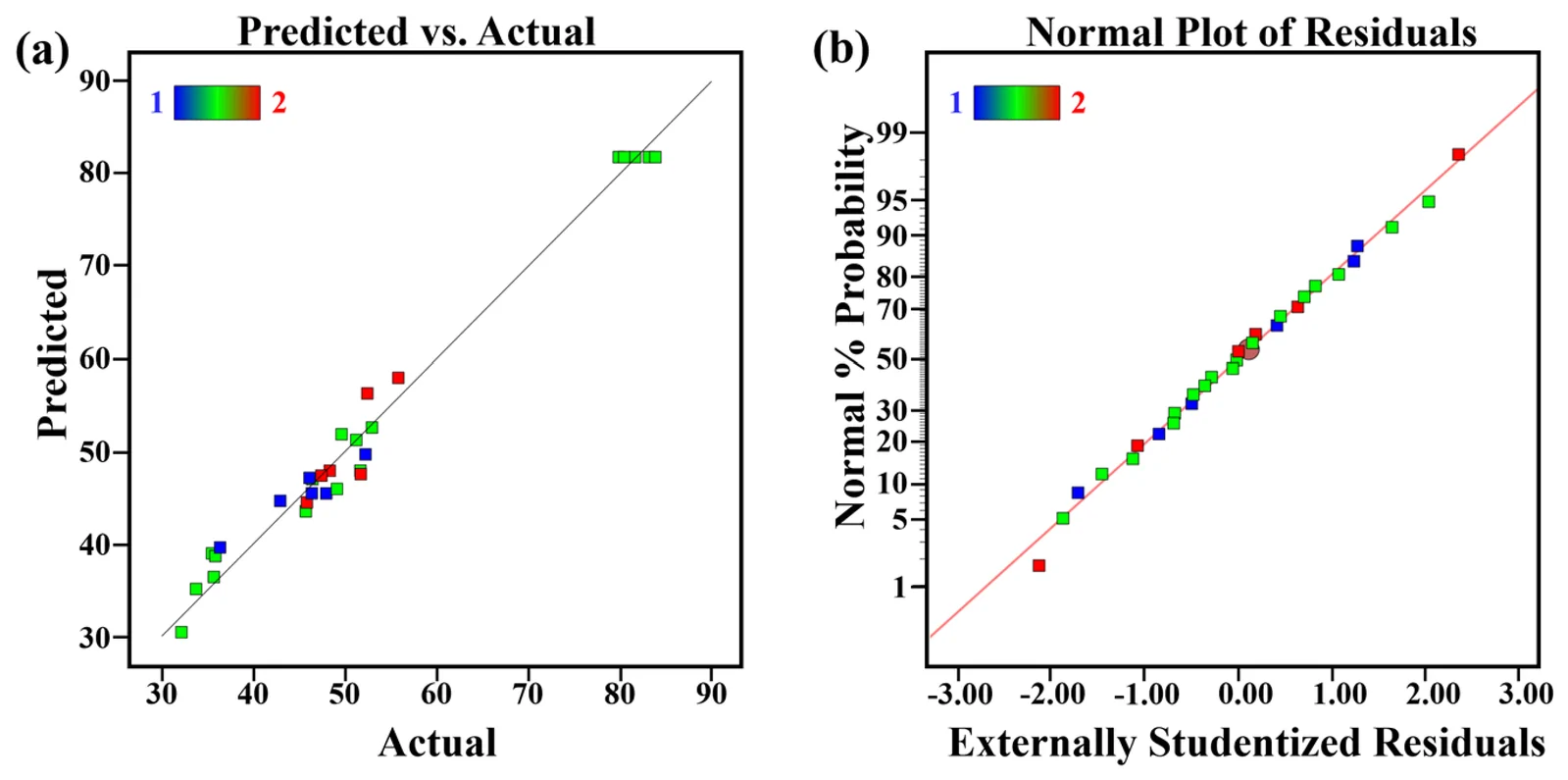
Model diagnostic plots. (**a**) Predicted vs. actual response values; (**b**) normal probability plot of residuals.

Figure 5a compares the model’s predicted values with the experimental measurements. The points lie close to a straight line, indicating strong agreement and high predictive accuracy. Figure 5b shows a normal probability plot of the residuals: the points cluster around the diagonal line with no clear deviation, suggesting that the residuals are approximately normally distributed and that the model’s assumptions are valid. The apparent overlap of data points near the zero residual (x ≈ 0) stems from the replicated center points of the response surface design. As shown in Table 3, analysis of variance of the regression model indicated that the equation was statistically significant and suitable for use. The model was highly significant (*p* < 0.0001), whereas the lack-of-fit term was not significant (*p* = 0.0848), suggesting that the model was appropriate for optimizing the target-size fraction. The coefficient of determination (R^2^) and adjusted coefficient of determination (adjusted R^2^) were 0.98 and 0.96, respectively, indicating good model fit and high reliability. These results support the use of the model to assess the effects of the predictive factors on cyst formation efficiency. The predicted R^2^ was 0.89, and its difference from the adjusted coefficient of determination was less than 0.2, indicating good agreement between the regression-predicted and experimental values and further supporting the feasibility of the model. Adequate precision reflects the noise signal ratio of the model, with values greater than 4 indicating an adequate signal. In this study, the adequate precision was 22.72, supporting the reliability of the regression equation. The coefficient of variation was 6.07%, further indicating good predictive performance and acceptable model feasibility.

Response surface methodology was used to evaluate the effects of multiple factors on the target-size fraction (Table 4). The model F value was 46.39, with *p* < 0.0001, indicating that the regression model was highly significant. Regression analysis of target-size fraction showed that the linear effects of Span 80 concentration (B) and Tween 80-CP concentration (C) in the continuous phase were not significant, whereas the quadratic and interaction terms were highly significant, indicating a nonlinear and synergistic effect of the two factors on target-size fraction. Span 80 (lipophilic) and Tween 80 (hydrophilic) act synergistically at the oil−–water interface. Together, they regulate the hydrophilic–lipophilic balance and promote the formation of a denser, more stable interfacial film. This strongly influences droplet formation and the final target-size fraction. The BC interaction showed that the ratio of Span 80 to Tween 80-cp was a key factor in encapsulation quality. This parameter should therefore be prioritized during process optimization. In addition, the quadratic terms (A^2^, B^2^, C^2^ and D^2^) for all four factors were highly significant (*p* < 0.0001). This indicates clear parabolic effects on target-size fraction and suggests that each factor had an optimal range. Values above or below this range reduced the target-size fraction. Notably, the BC interaction between Span 80 and Tween 80 in the continuous phase was also highly significant. This further suggests that their concentrations should be coordinated during process optimization to achieve optimal interfacial activity and stable droplet formation.

**Figure 6:**
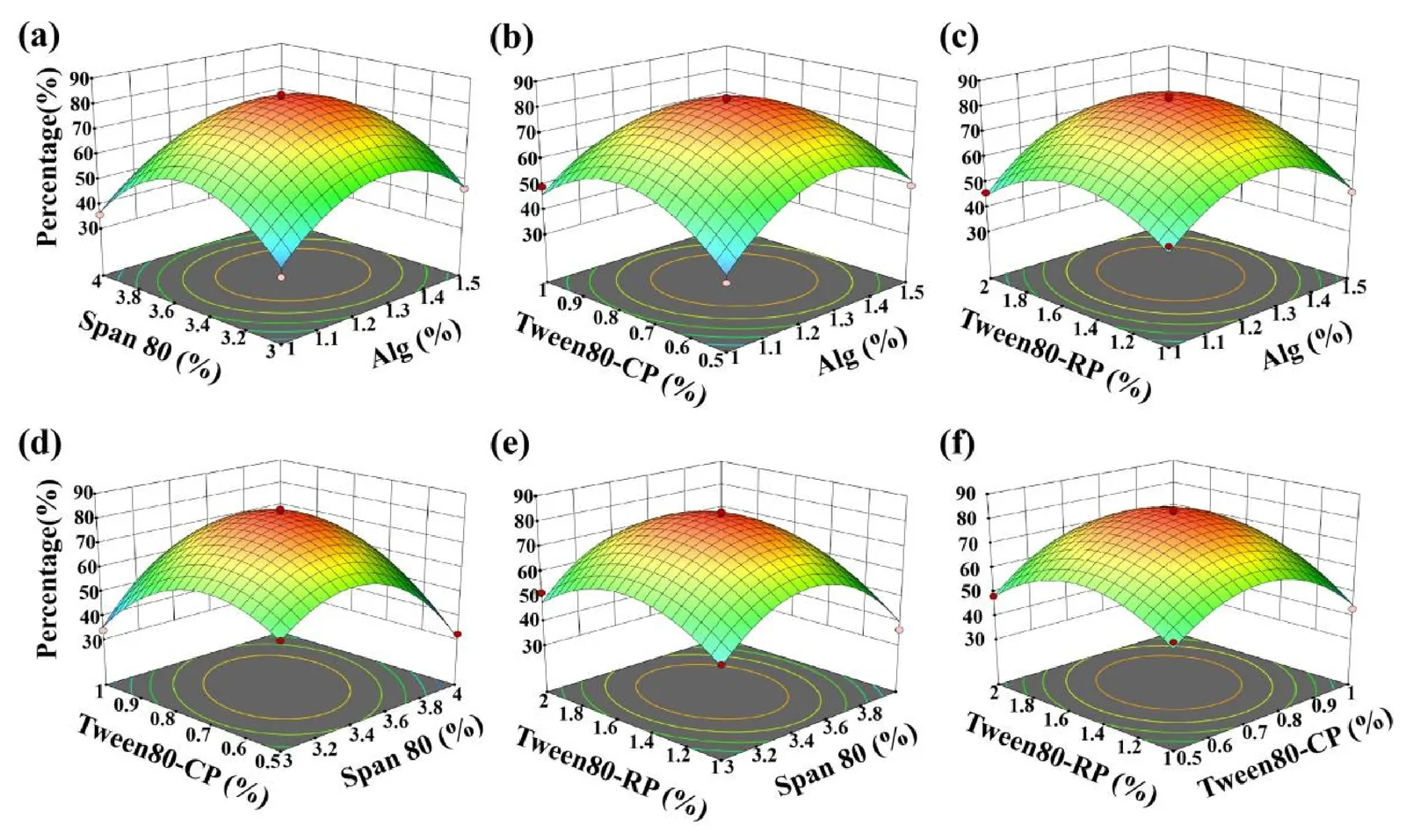
Response surface plots showing the interactions between (**a**) sodium alginate concentration and Span 80 concentration; (**b**) sodium alginate concentration and Tween 80 concentration; (**c**) sodium alginate concentration and Tween 80 concentration in the collection phase; (**d**) Span 80 concentration and Tween 80 concentration; (**e**) Span 80 concentration in the continuous phase and Tween 80 concentration; and (**f**) Tween 80 concentration in the continuous phase and Tween 80 concentration (The color gradient of the 3D response surface represents the magnitude of the response value (percentage). Blue corresponds to the lowest response value, which gradually increases through green and yellow, and reaches the maximum at red. The contour lines on the bottom plane adopt the same color scale to intuitively show the two-dimensional distribution of response values under different factor combinations).

The three-dimensional response surface plots show the relationships between the target-size fraction and any two factors. They reflect the main, interaction and quadratic effects. Steeper response surfaces and more elliptical contour lines indicate stronger interactions between the corresponding factors. As shown in Figure 6, the response surface for sodium alginate concentration and Tween 80-cp concentration in the continuous phase was steep (Figure 6b). The contour plot was distinctly elliptical. These features indicate a significant interaction between the two factors. A similar pattern was observed for Span 80 concentration and Tween 80-cp concentration, indicating a significant interaction (Figure 6d). Consistent with the analysis of variance in Table 4, these results suggest that the ratio of Span 80 to Tween 80, together with the synergistic effect of sodium alginate and Tween 80, plays an important role in regulating droplet interfacial stability and the final morphology of the microspheres. Response surface analysis confirmed the good fit of the model to the experimental data. It also revealed the effects of the key factors and their interactions on the target-size fraction. This provides a visual basis for precise control of subsequent process parameters.

In response surface analysis, the primary aim is to optimize the independent variables using the regression equation so as to determine the optimal process conditions for the target response. The model can be used to predict the response value, thereby reducing the experimental workload. To ensure its reliability and predictive accuracy, experimental validation is required. Accordingly, validation experiments were performed under the optimal conditions, and the feasibility and accuracy of the model were assessed by comparing the predicted values with the experimental results. Three parallel validation experiments were conducted under the optimal conditions (Table 5). The results showed that the mean experimental target-size fraction was 81.25%, in good agreement with the value predicted by the model. These findings indicate that the regression model accurately reflected the relationship between the factors and target-size fraction, and that the optimized conditions were feasible.

### 3.3. Cell Viability

The controlled preparation of calcium alginate microspheres (ALG microspheres) was successfully achieved through a microfluidic process optimized by response surface methodology. Based on this, the effects of microspheres prepared under the optimized conditions on cell viability were further assessed. The effect of microsphere co-culture under the optimized process conditions on the viability of pancreatic cancer cells was evaluated by live/dead staining. Calcein AM, used as a fluorescent probe, can penetrate the plasma membrane of viable cells and is hydrolyzed by intracellular esterases to generate green-fluorescent calcein in the cytoplasm. Cell viability can therefore be assessed by detecting the intensity of intracellular green fluorescence. Propidium iodide (PI) was used simultaneously as a marker of dead cells. Because PI cannot cross the membrane of viable cells, it selectively enters membrane-compromised dead cells and binds to DNA, thereby producing red fluorescence.

**Figure 7:**
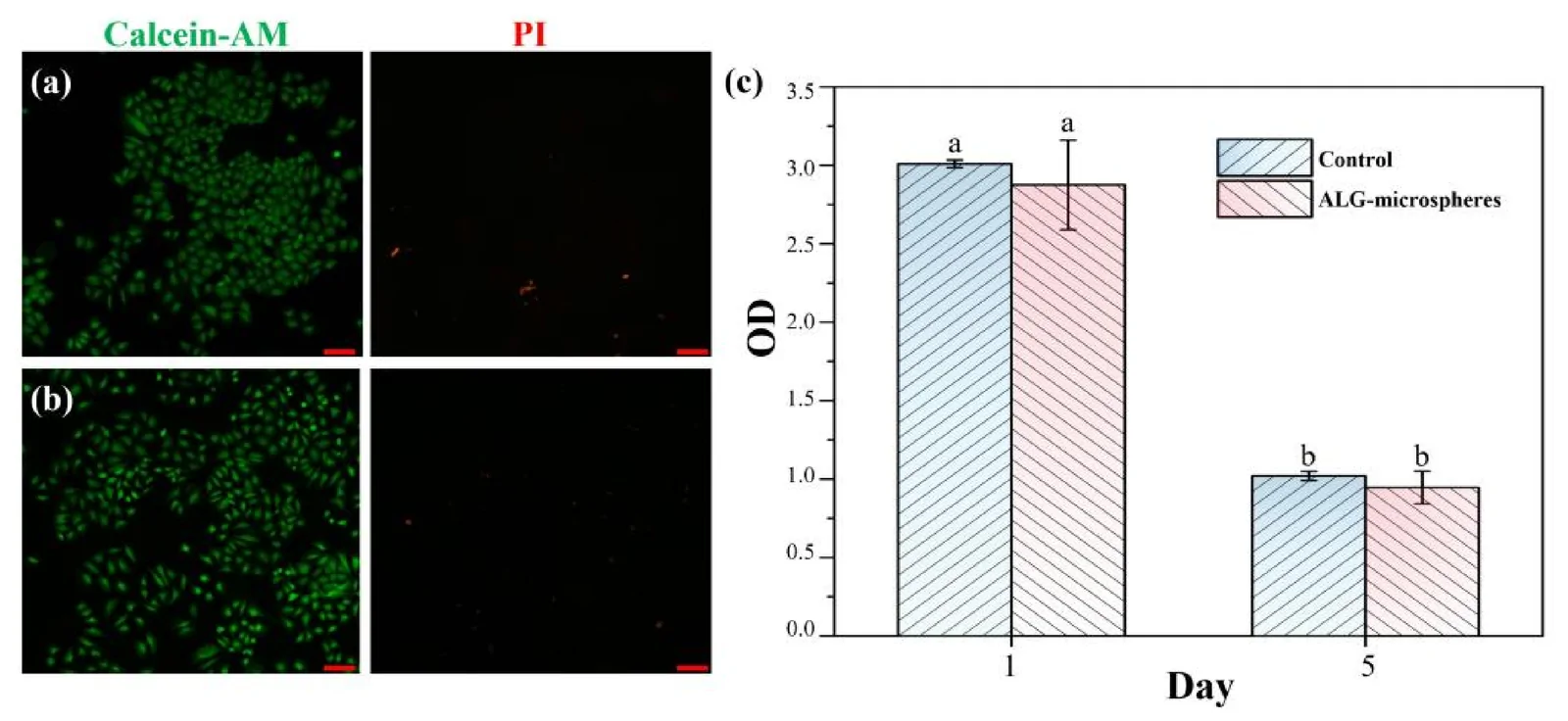
Effects of ALG microspheres on BxPC-3 cell viability. (**a**) Fluorescence images of BxPC-3 cells following co-culture with ALG microspheres and (**b**) the control group (without ALG microspheres), showing Calcein-AM staining (green), PI staining (red), scale bar 100 μm. (**c**) Cell viability determined by CCK-8 assay after 1 and 5 days of co-culture with ALG microspheres (columns with the same letter are not significantly different, *p* < 0.05. Data are presented as mean ± SD of 3 independent biological replicates, with 3 technical replicates per group).

Following staining with Calcein AM and PI, the cells were observed under an inverted fluorescence microscope, and the corresponding experimental images were collected. The results of the cell viability assay are shown in Figure 7a,b. The results showed that cells in the control group remained in good condition. Only a few PI-positive cells were observed. After co-culture with ALG microspheres prepared under the optimized conditions, only a small number of dead BxPC-3 cells were detected. Most cells remained viable. Both groups showed high cell viability, with no significant difference between them. To further validate these results, the viability of BxPC-3 cells co-cultured with ALG microspheres was evaluated by CCK-8 assay. As shown in Figure 7c, ALG microspheres prepared under the optimized conditions did not significantly affect BxPC-3 cell viability relative to the cell-only control group. No obvious cytotoxicity was observed, indicating good cytocompatibility and biological safety.

**Figure 8:**
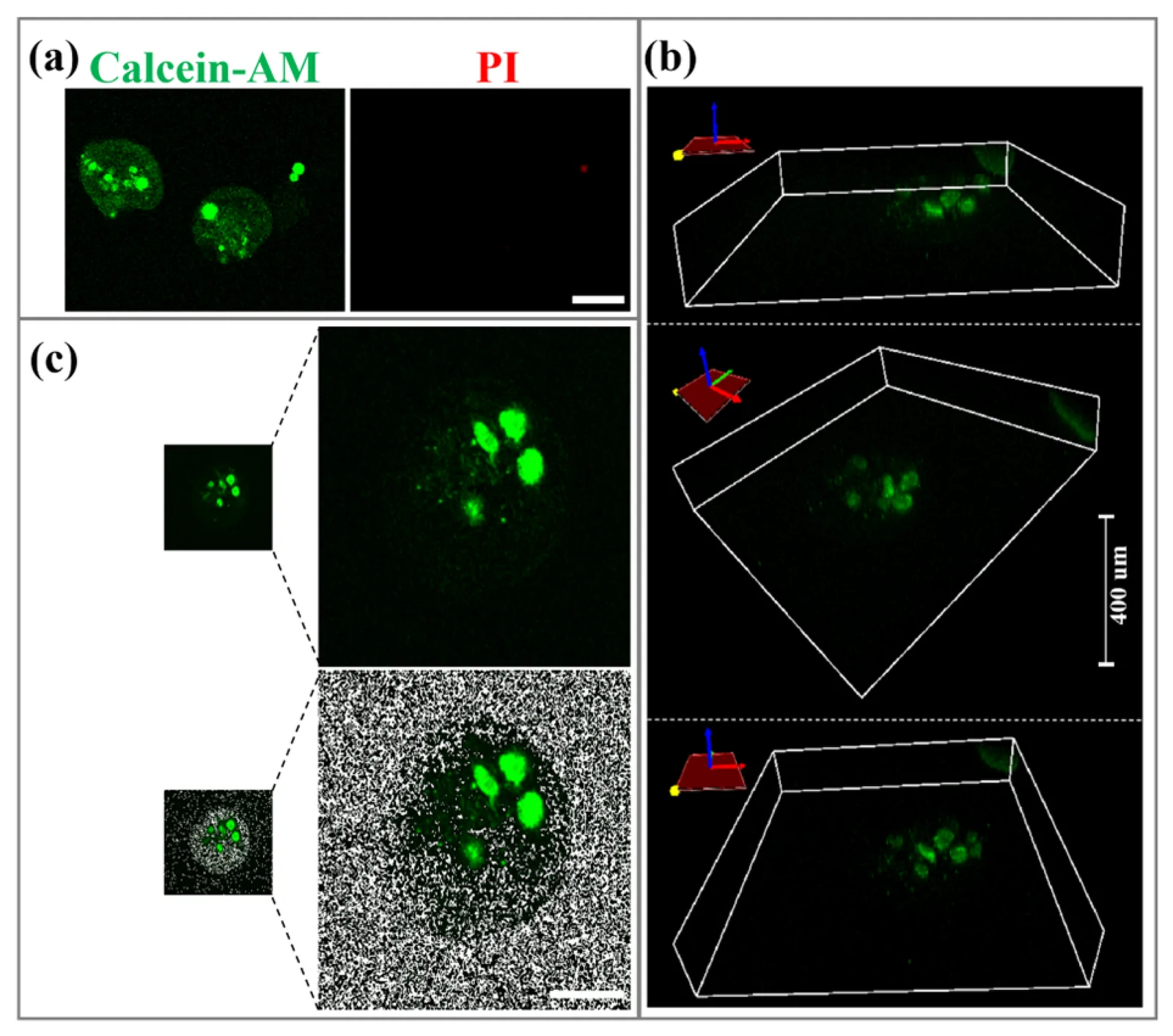
Characterization of BxPC-3 cells encapsulated in calcium alginate microspheres by confocal laser scanning microscopy, scale bar 100 μm. (**a**) Live/dead staining. (**b**) Three-dimensional morphology of cells within the microspheres, scale bar 400 μm. (**c**) Three-dimensional reconstructed images, scale bar 100 μm.

To evaluate the growth status of BXPC-3 cells within calcium alginate microspheres, together with the toxicity and biocompatibility of the microspheres toward the encapsulated cells, the 3D cell system was analyzed by confocal laser scanning microscopy and calcein AM/PI double staining. As shown in Figure 8a, numerous bright and uniform green fluorescent signals were observed in the calcein AM channel, and the cells displayed three-dimensional clustered growth with uniform fluorescence intensity. Only a few sporadic red fluorescent signals were detected in the PI channel, indicating a very low proportion of dead cells within the microspheres. BXPC-3 cells prepared by the microfluidic method maintained high viability, further confirming the absence of cytotoxicity and the good biocompatibility of the calcium alginate microspheres prepared in this study. Figure 8b shows that BXPC-3 cell clusters encapsulated by the microfluidic method exhibited intact morphology, with closely packed cells forming clusters of 30–50 μm for 3 days. The clear green fluorescence indicated sustained viability, suggesting that the hydrogel network of the calcium alginate microspheres did not cause mechanical damage. The encapsulated pancreatic cancer cells retained intact cellular architecture and normal morphological characteristics, indicating preservation of cellular structure. In addition, stained calcium alginate microspheres encapsulating BXPC-3 cells were scanned three-dimensionally and reconstructed from multiple perspectives to assess the spatial distribution of cell clusters within the microspheres (Figure 8c). Reconstructed images from multiple perspectives showed that green fluorescent signals were concentrated within the three-dimensional space of the microspheres as clustered aggregates. BXPC-3 cells encapsulated in the alginate matrix formed dense three-dimensional cell clusters after culture, with fluorescence signals mainly located within the interior of the construct and showing clear spatial aggregation from different viewing angles. These findings indicate three-dimensional cell aggregation and assembly within the hydrogel rather than two-dimensional adherent growth. As a mild three-dimensional scaffold, alginate hydrogel can partially restrict disordered cell diffusion, provide a relatively stable external support environment, and promote cluster growth.

## 4. Conclusions

In conclusion, a microfluidic approach for the controlled preparation of calcium alginate microspheres was successfully established and optimized using response surface methodology. The results showed that sodium alginate concentration and the surfactant composition of the continuous and receiving phases were key factors affecting target-size fraction and morphology. Response surface analysis further demonstrated that the interactions between Span 80 and Tween 80-CP, as well as between sodium alginate and Tween 80-RP, significantly influenced interfacial stability and microsphere formation. The regression model showed a good fit and satisfactory predictive ability, and validation experiments confirmed the feasibility of the optimized process, with a target-size fraction of 81.25% in the validation experiments.

Moreover, the optimized calcium alginate microspheres showed favorable biocompatibility. Live/Dead staining and CCK-8 analysis indicated that the microspheres exhibited no obvious cytotoxicity and did not significantly affect the viability of BxPC-3 cells. Confocal imaging further showed that the encapsulated cells remained high viability and formed compact three-dimensional clusters within the alginate matrix, indicating that the hydrogel network provided a favorable microenvironment for tumor cell aggregation and growth. Taken together, these findings suggest that the optimized microfluidic alginate encapsulation system is a promising and biocompatible platform for constructing three-dimensional in vitro models of pancreatic cancer. This system may also have potential in biomedical applications such as cell encapsulation and drug delivery, and may provide a methodological basis for the development of cell carriers in tissue engineering and regenerative medicine.

## Figures and Tables

**Table 1:** Factors and levels of the Box–Behnken experimental design.

Factors	Level		
	−1	0	1
A Sodium alginate concentration/(%)	1	1.25	1.5
B Span 80 concentration/(%)	3	3.5	4
C Tween 80-CP concentration/(%)	0.5	0.75	1
D Tween 80-RP concentration/(%)	1	1.5	2

**Table 2:** Box–Behnken experimental design and corresponding results.

	Factor 1	Factor 2	Factor 3	Factor 4	Response
Number	A (%)	B (%)	C (%)	D (%)	R (%)
1	1	3.5	0.75	2	45.83
2	1.25	4	1	1.5	51.23
3	1.5	3.5	0.5	1.5	49.62
4	1.25	3.5	0.5	2	48.33
5	1	3.5	0.75	1	47.95
6	1.5	3.5	0.75	1	46.12
7	1	3.5	1	1.5	49.12
8	1.25	3.5	0.75	1.5	79.86
9	1.25	3.5	1	2	52.43
10	1.25	3	0.75	2	51.72
11	1.5	4	0.75	1.5	45.72
12	1.25	3.5	0.75	1.5	81.67
13	1.25	3.5	0.75	1.5	80.45
14	1	3.5	0.5	1.5	35.85
15	1.5	3	0.75	1.5	46.43
16	1.25	3	0.5	1.5	52.94
17	1.25	3.5	0.75	1.5	83.15
18	1.25	4	0.75	2	47.42
19	1.5	3.5	0.75	2	55.82
20	1.25	3.5	0.75	1.5	83.86
21	1.25	3.5	0.5	1	52.24
22	1.25	3.5	1	1	42.93
23	1.25	3	0.75	1	46.37
24	1	3	0.75	1.5	35.48
25	1.25	4	0.75	1	36.36
26	1	4	0.75	1.5	35.68
27	1.5	3.5	1	1.5	51.67
28	1.25	4	0.5	1.5	32.15
29	1.25	3	1	1.5	33.73

A: Sodium alginate concentration, B: Span 80 concentration, C: Tween 80-1 concentration, D: Tween 80-2 concentration, and R: target-size fraction.

**Table 3:** Model summary statistics of the quadratic response surface.

Responses	Models						
	F-Value	Prob > F	R^2^	Adjusted R^2^	Predicted R^2^	Adeq. Prec.	C.V. (%)
R	46.39	< 0.0001	0.98	0.96	0.89	22.72	6.07

Adjusted R^2^, adjusted R^2^; Adeq. prec., adequate precision; C.V., coefficient of variation. Underlined values indicate significant models (*p* < 0.05).

**Table 4:** Effects of individual factors in the regression equation and analysis of variance results.

Factor	Factor Effect	F-Value	*p*-Value
		46.39	<0.0001
A	+3.79	17.42	0.0009
B	−1.51	2.76	0.1186
C	+0.8317	0.8394	0.3751
D	+2.47	7.37	0.0167
AB	−0.2275	0.0209	0.8870
AC	−2.80	3.18	0.0961
AD	+2.96	3.53	0.0812
BC	+9.57	37.07	<0.0001
BD	+1.43	0.8243	0.3793
CD	+3.35	4.55	0.0512
A^2^	−18.29	219.32	<0.0001
B^2^	−22.04	318.72	<0.0001
C^2^	−17.42	198.98	<0.0001
D^2^	−14.76	142.84	<0.0001

AB, AC, AD, BC, BD, and CD represent the interaction effects of factors A, B, C, and D; A^2^, B^2^, C^2^, and D^2^ are the squared effects of the factors; underlined values indicate significant factors (*p* < 0.05).

**Table 5:** Comparison of predicted and experimental R values at randomly selected test points under specific conditions.

Checkpoint Conditions A/B/C/D	R (%) Target-Size Fraction		
	Pred. Data	Exp. Data	Agree (%)
1.28/3.49/0.75/1.55	82.15%	81.25 ± 0.84%	98.89%

## Data Availability

The raw data supporting the conclusions of this study will be made available by the corresponding authors upon reasonable request.
